# SPEEK Membrane of Ultrahigh Stability Enhanced by Functionalized Carbon Nanotubes for Vanadium Redox Flow Battery

**DOI:** 10.3389/fchem.2018.00286

**Published:** 2018-07-26

**Authors:** Mei Ding, Xiao Ling, Du Yuan, Yuanhang Cheng, Chun Wu, Zi-Sheng Chao, Lidong Sun, Chuanwei Yan, Chuankun Jia

**Affiliations:** ^1^College of Materials Science and Engineering, Changsha University of Science and Technology, Changsha, China; ^2^Institute of Metal Research, Chinese Academy of Sciences, Shenyang, China; ^3^Department of Materials Science and Engineering, Faculty of Engineering, National University of Singapore, Singapore, Singapore; ^4^State Key Laboratory of Mechanical Transmission, School of Materials Science and Engineering, Chongqing University, Chongqing, China; ^5^Key Laboratory of Advanced Energy Materials Chemistry (Ministry of Education), Nankai University, Tianjin, China

**Keywords:** redox flow battery, vanadium redox flow battery, ion exchange membrane, sulfonated poly(ether ether ketone), carbon nanotube

## Abstract

Proton exchange membrane is the key factor of vanadium redox flow battery (VRB) as their stability largely determine the lifetime of the VRB. In this study, a SPEEK/MWCNTs-OH composite membrane with ultrahigh stability is constructed by blending sulfonated poly(ether ether ketone) (SPEEK) with multi-walled carbon nanotubes toward VRB application. The carbon nanotubes disperse homogeneously in the SPEEK matrix with the assistance of hydroxyl group. The blended membrane exhibits 94.2 and 73.0% capacity retention after 100 and 500 cycles, respectively in a VRB single cell with coulombic efficiency of over 99.4% at 60 mA cm^−2^ indicating outstanding capability of reducing the permeability of vanadium ions and enhancing the transport of protons. The ultrahigh stability and low cost of the composite membrane make it a competent candidate for the next generation larger-scale vanadium redox flow battery.

## Introduction

Vanadium redox flow battery (VRB) is one of the most promising large-scale energy storage technologies with the advantages of decoupled energy storage and power output, flexible design, long lifetime, and so on (Li et al., 2011; Skyllas-Kazaocs et al., 2011; Weber et al., 2011; Yang et al., 2011; Wang et al., 2013). Nevertheless, the commercialization of the VRB is still hindered by low stability and high cost of the membrane (Jia et al., 2014; Noorden, 2014; Zhang, 2014), which is the key component to separate the catholyte and anolyte and to transport protons (Jiang et al., 2016; Yu and Xi, 2016). An ideal membrane is expected to bear high chemical stability, good mechanical strength, high proton conductivity, and low vanadium ions crossover between anolyte and catholyte to suppress the imbalance of electrolyte. To date, the Nafion membranes are widely used in the VRB systems because of their high proton conductivity and excellent chemical stability (Reed et al., 2015; Teng et al., 2015). However, the high cost and high vanadium ion permeability deteriorate the battery performance, and thus are unsuitable for large-scale applications and future commercialization (Xi et al., 2007; Luo et al., 2013; Kima et al., 2014; Lin et al., 2015). Recently the sulfonated poly (ether ether ketone) (SPEEK) membrane is considered as a substitution to the Nafion, in view of its low cost (Ling et al., 2012; Liu et al., 2014) and high proton conductivity (Jia et al., 2012; Wei et al., 2012). Nonetheless, the SPEEK membrane suffers from noticeable vanadium crossover in light of the high degree of sulfonation, giving rise to low mechanical/chemical stability and hence short cycling lifetime of VRB. Many reports have reported that blending the high sulfonated SPEEK membrane with materials lowers its crossover of vanadium ions (Wang et al., 2012; Dai et al., 2014a; Li et al., 2014). However, the mechanical and chemical stabilities of these blended SPEEK membranes are still unable to meet the long-term cycling requirements of VRB. On the other hand, blended SPEEK membrane with graphene and carbon nanotubes exhibits better stability compared to the membrane blended with other materials (Dai et al., 2014b; Jia et al., 2015a). The single VRB cell with SPEEK/graphene composite membrane reported by Dai et al. exhibited a capacity retention of 56.3% upon 300 cycles (Dai et al., 2014b). Our previous work reported a reinforced mechanical strength of the SPEEK/SCCT composite membrane for VRB (Jia et al., 2015a). However, until now, to the best of our knowledge, ultra-long lifetime (>1,000 cycles) in SPEEK based membranes has been a critical but unaddressed issue for future commercialization of VRBs.

Herein, we report a novel SPEEK membrane enhanced by hydroxylated multi-walled carbon nanotubes (SPEEK/MWCNTs-OH). The composite membrane exhibits high mechanical strength and low vanadium ions permeability, owing to the reinforcement effect of SPEEK and MWCNTs-OH. The proton conductivity is promoted, since the OH groups on MWCNTs form additional channels for proton transport. The VRB single cells based on the SPEEK/MWCNTs-OH membrane show an ultralong cycling lifetime (94.2% capacity retention after 100 cycles, 73.0% capacity retention after 500 cycles) and ultrahigh Coulombic efficiency (>99.4%). The current study contributes to the design of advanced membranes for efficient VRB toward large-scale energy storage applications.

## Experimental section

Poly(ether ether ketone) (Victrex, PEEK 450 PF), Dimethylsulfoxide (DMSO) and sulfuric acid (98 wt.%) were purchased from Sinopharm Chemical Reagent Co. Ltd. The MWCNTs-OH (length: 0.5–2 mm, diameter: <8 nm, purity: >95%, OH content: 5.58 wt.%) were purchased from Nanjing XFNANO Materials Tech Co. Ltd. All chemicals were used as purchased without further purification.

The sulfonation process of PEEK was described in our previous work (Jia et al., 2010). The degree of sulfonation for the SPEEK was determined by titration, about 50.2% in this study. In order to enhance the mechanical property of the SPEEK matrix while keep its low price, low electron conductivity, a low MWCNTs-OH concentration (0.7 wt%) was chosed in this work. The SPEEK/MWCNTs-OH membrane was prepared sequentially by the steps as follows: (1) 1.7 g SPEEK was dissolved in 40 mL DMSO, resulting a SPEEK solution; (2) 12 mg MWCNTs-OH was added into the above SPEEK solution under magnetic stirring; (3) the resulting solution was stirred for more than 5 h and subsequently sonicated for 10 min at room temperature; (4) the SPEEK/MWCNTs-OH solution was cast onto a home-made glass mold and heated at 100°C for 15 h in an oven until all of DMSO being evaporated; (5) the resultant membrane was cooled down to room temperature and peeled off from the glass mold. The thus-obtained SPEEK/MWCNTs-OH membrane (thickness is 90 μm) was stored in deionized water before its characterization and application in VRB.

The surface morphology of the membrane was examined by scanning electron microscopy (SEM, ZEISS). The membrane was sputtered with gold prior to SEM observation. The mechanical property of the membrane was measured using a CMT 6502 tension tester (Shenzhen Instron Corporation China). The tensile strength of membrane was calculated using the equation described in literature as follows (Ling et al., 2012):

$$\begin{array}{l} \text{Mechanical strength}={\text{S}}_{\text{m}}/\left(\text{W}\times \text{L}\right) \end{array}$$

Where S_m_ is the maximum strength of membrane, W is the width of different membrane samples and L is thickness of different membranes.

The permeability of VO^2+^ ions across the membrane was measured according to our previous method (Jia et al., 2010, 2015a). Briefly, a redox flow single cell was filled up with two kinds of electrolytes in respective reservoirs, i.e., 70 mL of 1.5 M VOSO_4_ in 2.0 M H_2_SO_4_ solution and 70 mL of 1.5 M MgSO_4_ in 2.0 M H_2_SO_4_. Solution of 1 mL was sampled from the MgSO_4_ compartment at certain time interval and was measured with the UV-vis spectrometer. The solutions in both reservoirs were continuously stirred to avoid concentration polarization. The permeability was calculated using the following equation:

$$\begin{array}{l} {\text{V}}_{\text{B}}\frac{\text{d}{\text{C}}_{\text{B}}\left(\text{t}\right)}{\text{dt}}=\text{A}\frac{\text{P}}{\text{L}}\left({\text{C}}_{\text{A}}-{\text{C}}_{\text{B}}\left(\text{t}\right)\right) \end{array}$$

where V_B_ is the volume of MgSO_4_ in the reservoir, C_B_(t) is the concentration of ${\text{VO}}_{2}^{+}$ ions in the MgSO_4_ compartment as a function of time t, C_A_ is the ${\text{VO}}_{2}^{+}$ concentration in the VOSO_4_ compartment, A is the active area of the membrane (28 cm^2^), L is the thickness of the membrane, and P is the permeability of V (IV) ions.

A single cell with 1.5 M VOSO_4_ and 2 M H_2_SO_4_ as the catholyte (80 mL) and anolyte (80 mL), respectively, was employed to determine the area resistance (R) of the membranes by DME-20 Battery Internal Resistance Tester. The area resistance and conductivity (σ) of the membranes were computed as:

$$\begin{array}{l} \text{R}=\left({\text{R}}_{1}-{\text{R}}_{2}\right)\times \text{A} \end{array}$$

and σ = L/R

where R_1_ and R_2_ represent the resistances of the single cell with and without SPEEK/MWCNTs-OH, respectively.

Galvanostatic intermittent titration technique (GITT) measurement was conducted in a VRB single cell to further study the area resistance and the permeation of vanadium ions through the membranes, as detailed in reference (Jia et al., 2015b). In this test, 25 mL 1.5 M V^2+^/V ^3+^ in 2.0 M H_2_SO_4_ and 25 mL 1.5 M VO^2+^/${\text{VO}}_{2}^{+}$ in 2.0 M H_2_SO_4_ solutions were cycled through anodic and cathodic reservoirs, respectively. The GITT measurements were carried out ranging from 0.7 to 1.65 V. The cell was charged and discharged at a current density of 40 mA cm^−2^ for 4 min, followed by an open circuit relaxation for 4 min.

The open circuit voltage (OCV) of the VRB single cell was monitored at room temperature after charged to a charge state of 75%.

The VRB single cell was assembled with two carbon felt electrodes, two conductive plastic plate current collectors, membrane and steel endplate. The active area is 28 cm^2^. 25 mL 1.5 M V^2+/^V^3+^ in 2.0 M H_2_SO_4_ and 25 mL 1.5 M VO^2+^/${\text{VO}}_{2}^{+}$ in 2.0 M H_2_SO_4_ were used as anolyte and catholyte, respectively. The VRB single cell was charged and discharged at a constant current density of 50 mA cm^−2^ with the voltage ranging from 0.7 to 1.65 V. After around 380 cycles, the compartments were refilled with fresh electrolytes and the carbon felt electrodes were replaced with new pieces. The rate performance was studied at a current density ranging from 40 to 120 mA cm^−2^ and then back to 80 mA cm^−2^ for cycling.

## Results and discussion

**Figure 1:**
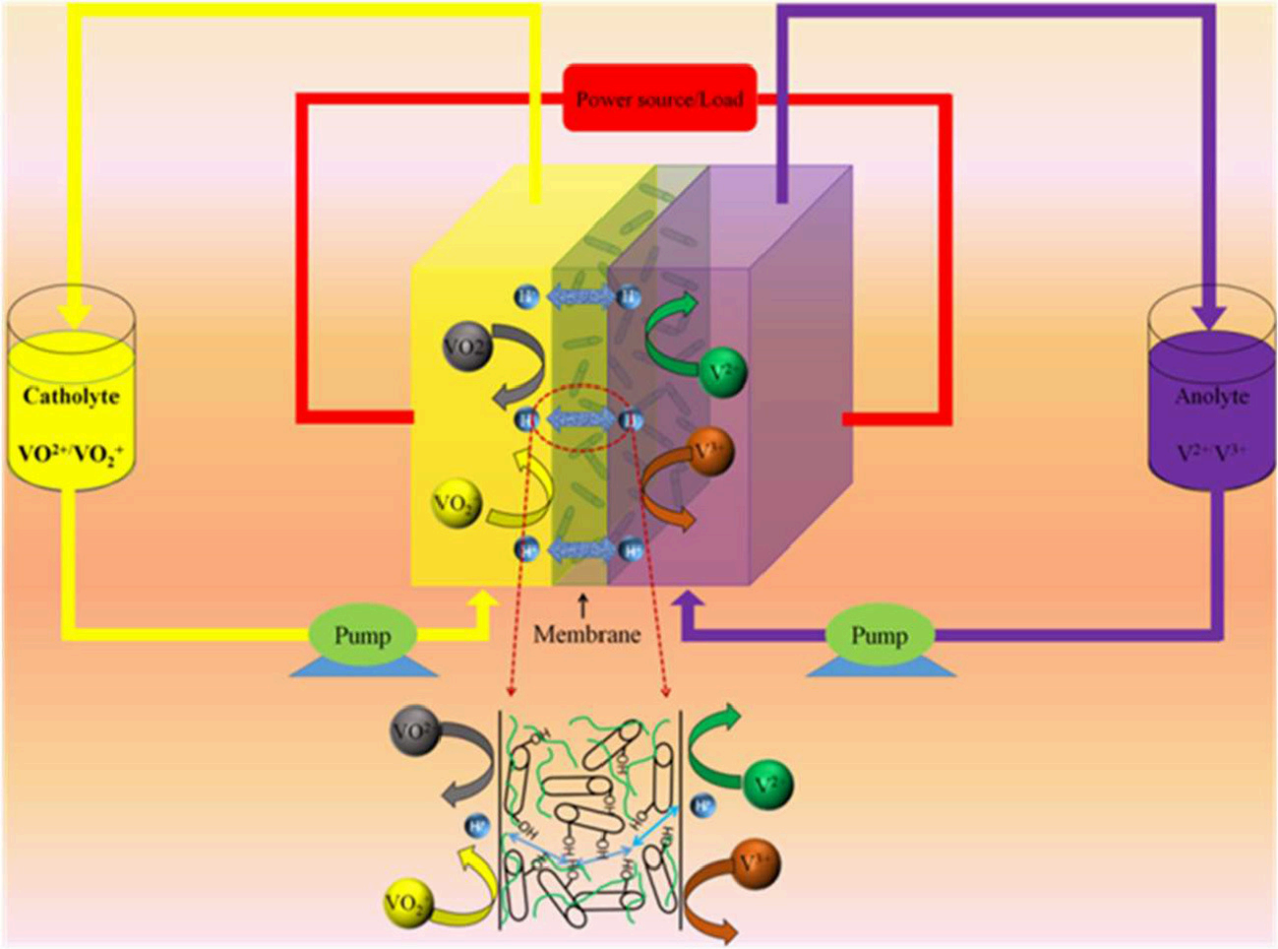
Schematic illustration of vanadium redox flow battery employing the SPEEK/MWCNTs-OH composite membrane.

The mechanical property of the membranes is of first concern in view of the long-term stability and service for a VRB system. It has been determined that the mechanical strength increases upon blending with the MWCNTs-OH from 40.1 to 61.8 MPa. This can be attributed to the reinforcement from the carbon nanotubes, which are of high mechanical strength and behaves as strengthening component in the composite. The hydroxyl groups that graft on the surface play an important role in forming the composite structure, making the nanotubes bonded strongly to the SPEEK matrix, as illustrated in Figure 1.

**Figure 2:**
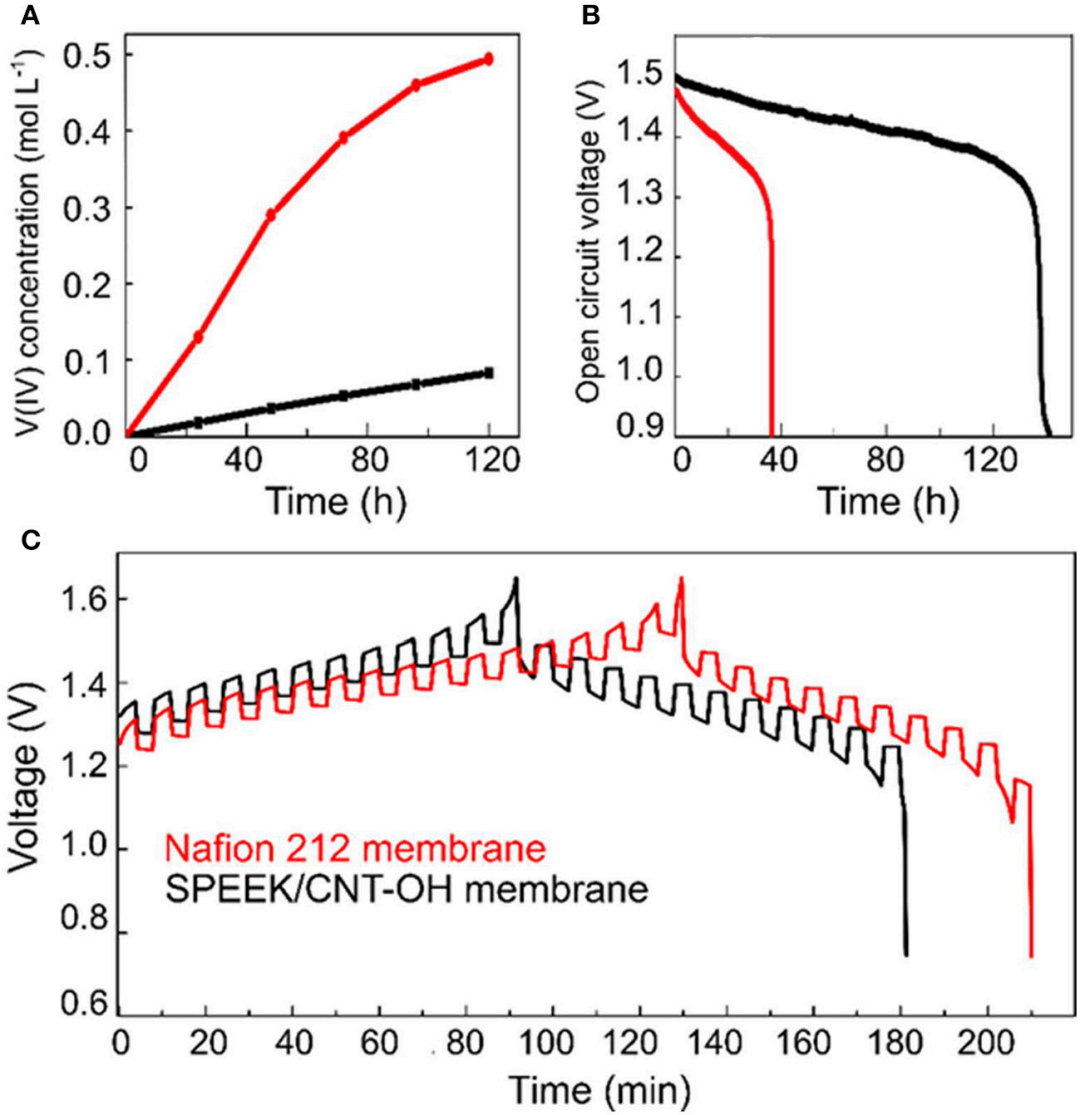
Comparison between Nafion 212 (red) and SPEEK/MWCNTs-OH (black) membranes: **(A)** VO^2+^ permeability; **(B)** Open circuit voltage decay; **(C)** GITT test.

The permeability of SPEEK membranes is highly suppressed by blending with the carbon nanotubes, which is even better than commercial Nafion 212. As can be seen from Figure 2A that the concentration of VO^2+^ ions across the SPEEK/MWCNTs-OH membrane is much lower than the Nafion 212, about 10-fold less at 120 h. The permeability of VO^2+^ ions is thus computed to be about 1.93 × 10^−7^ cm^2^ min^−1^ for the SPEEK/MWCNTs-OH membranes, over 4 times lower than that of Nafion 212 (8.23 × 10^−7^ cm^2^ min^−1^). The permeation of vanadium ionic species (cathode, ${\text{VO}}_{2}^{+}$/VO^2+^; anode, V^3+^/V^2+^) across the membrane leads to a severe self-discharge of the battery and in turn decay of the open circuit voltage (OCV). Figure 2B compares the OCV decay of the VRB cells with different membranes, where the cells were initially charged to 75% state of capacity. Both of the decay curves gradually decrease until a drastic drop appears. The retention time over 1.3 V in SPEEK/MWCNTs-OH membrane is about 100 h longer than that of Nafion 212. This further confirms the superior ability of the SPEEK/MWCNTs-OH membrane in suppressing vanadium ion crossover, consistent with the permeability test. In general, the crossover becomes more serious when the cells are under intermittent charging-discharging process. Figure 2C displays a longer retention time of the discharge potential for SPEEK/MWCNTs-OH membrane as compared to that of Nafion 212, in good agreement with the above discussion. Consequently, the composite membrane of SPEEK/MWCNTs-OH suppresses the permeability of vanadium ions. This can be attributed to the additives of carbon nanotubes that block the diffusion of bulky vanadium ions.

**Figure 3:**
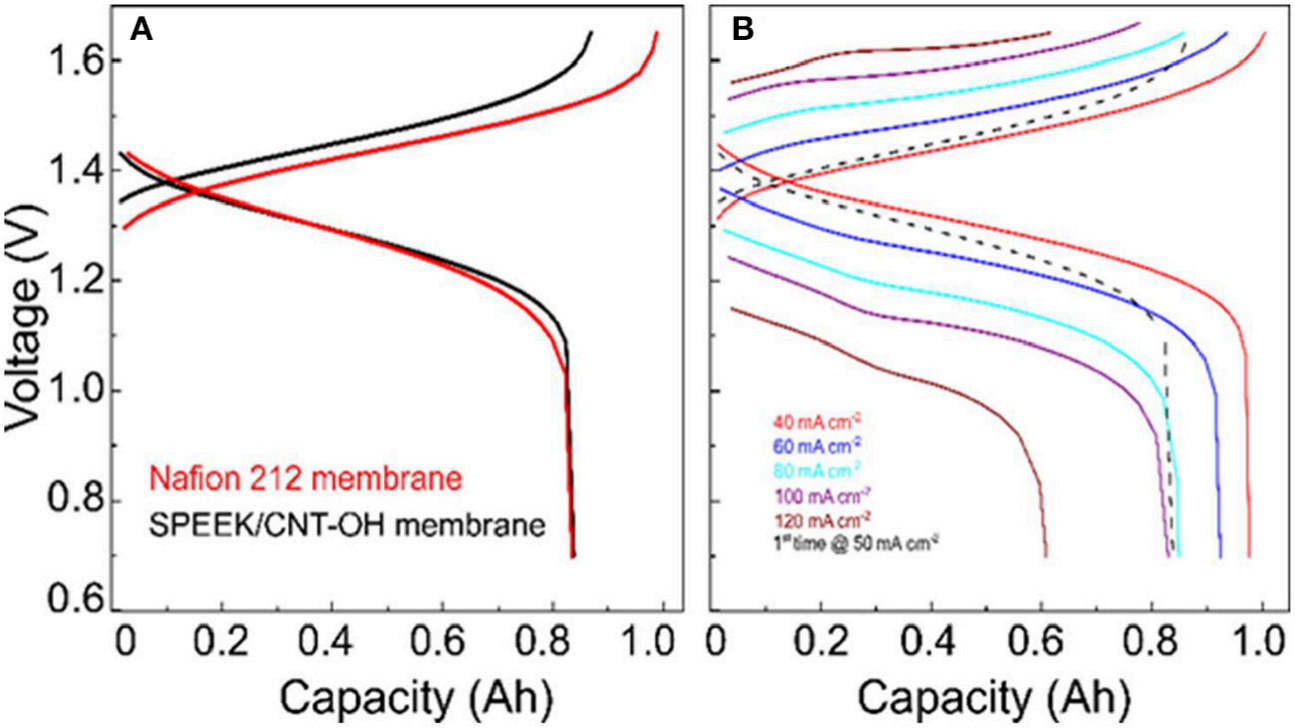
Charge-discharge curve of VRB with SPEEK/MWCNTs-OH membranes: **(A)** compared with the Nafion 212 membrane at 50 mA cm^−2^; **(B)** tested under different current densities.

The charge-discharge curves of VRB cells with SPEEK/MWCNTs-OH and Nafion 212 membrane are compared in Figure 3A. It clearly shows that the discharge capacity of cells with SPEEK/CNTs-OH membrane is larger than that with Nafion 212, thereby giving rise to a higher Columbic efficiency (CE). This is mainly due to the lower permeability and thus lower self-discharge. Meanwhile, a relatively higher and lower average charge and discharge voltage for cells with SPEEK/MWCNTs-OH membranes are observed respectively, because of the large IR drop resulted from the high area resistance (1.04 vs. 0.6 Ω cm^2^ for SPEEK/MWCNTs-OH and Nafion 212 membranes Jia et al., 2012, respectively). The corresponding IR drop is around 70 and 55 mV, in accordance with the GITT results (see Figure 2C). The discharge capacity of the VRB single cell with SPEEK/MWCNTs-OH membrane is larger than 0.6 A h (>60% of the initial discharge capacity at 40 mA cm^−2^) even at a current density of 120 mA cm^−2^, indicating a good rate performance (Figure 3B).

**Figure 4:**
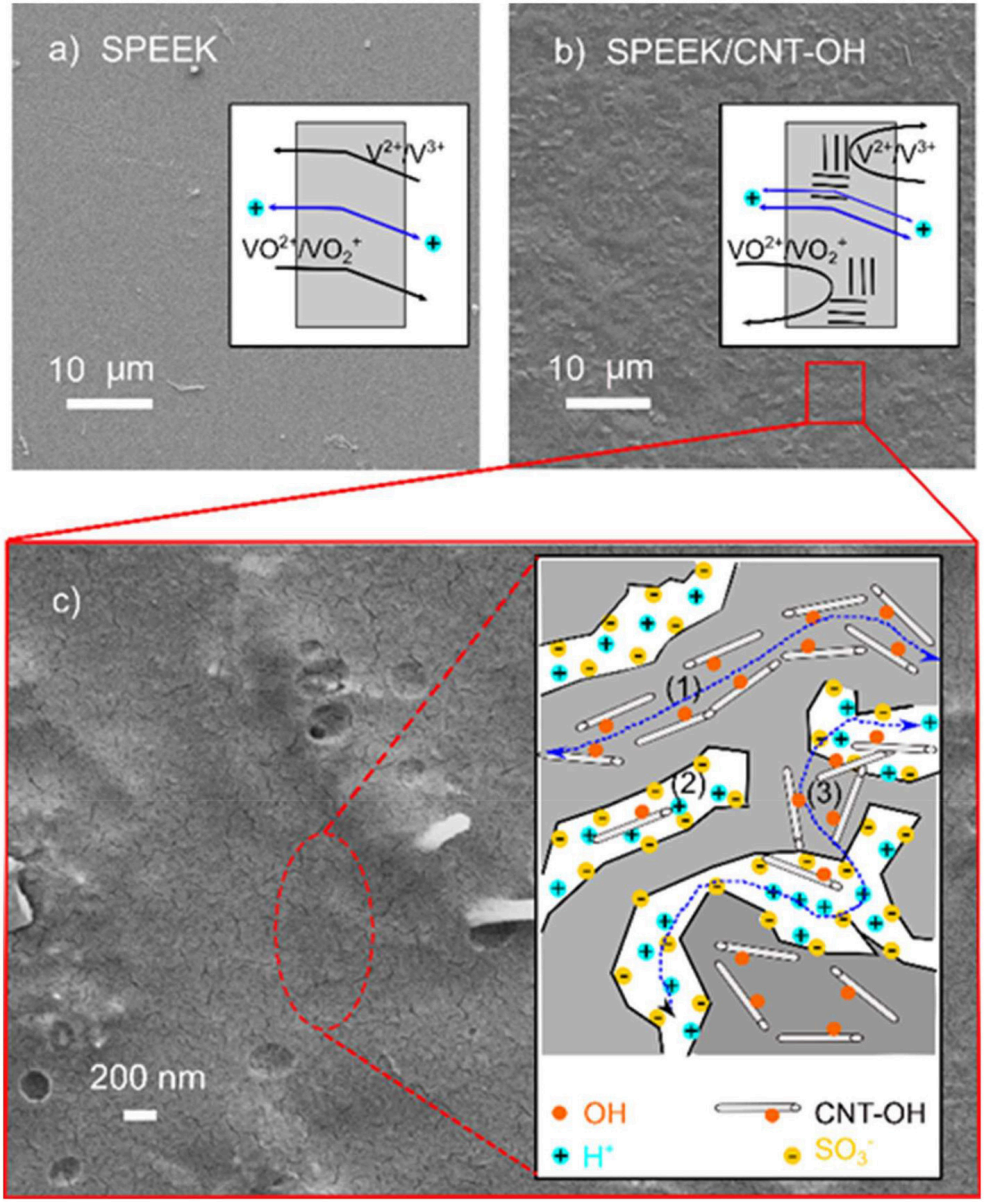
Top-view SEM images of different membranes: **(a)** pure SPEEK membrane; **(b,c)** SPEEK/MWCNTs-OH composite membrane; the inset in **(c)** is an illustration showing the interaction between MWCNTs-OH and SPEEK polymer matrix.

High Columbic efficiency (CE) in the VRB cell indicates low capacity loss in a charge-discharge cycle, as the CE is the ratio of discharge to charge capacity (He et al., 2015; Jia et al., 2015a). The capacity loss mainly arises from permeability of vanadium ions, which is in turn closely related to the membrane structure. The SPEEK/MWCNTs-OH membrane shows no significant phase separation while presents much rougher surface as compared to the original SPEEK membrane, as displayed in Figures 4a,b. It reveals that small clustering of carbon nanotubes are embedded uniformly in the SPEEK matrix, therefore increasing the surface roughness and decreasing the pathway for ion diffusion. Besides, the carbon nanotubes behave as extra barriers to vanadium ions. Therefore, the composite membrane suppresses the permeability of vanadium ions. More interesting, the proton conductivity is also enhanced with the composite structure (about 10.1 mS cm^−1^, 1.5-fold higher than that of SPEEK membrane). Detailed examination discloses worm-like structures (>500 nm in length) on the surface of composite membrane, as shown in Figure 4c. This can be assigned to the traces of carbon nanotubes (500–2,000 nm in length used in this study) that bond to the SPEEK matrix. Generally, the carbon nanotubes play the following roles in the SPEEK polymer matrix (see the inset in Figure 4c): (1) the nanotubes are aligned to the SPEEK backbones, forming protective layers to reduce the corrosion of aromatic backbones in a VRB cell; (2) the nanotubes increase the tortuosity of ionic channels developed by sulfonic acid groups (S-channel); (3) the hydroxyl groups at the nanotube surface form new ionic channels (C-channel) and hence facilitate proton transport (He et al., 2015); (4) the carbon nanotubes bridge between the clusters and further contribute to the proton transport (Tunuguntla et al., 2016). Therefore, with the combination of reduced permeability of vanadium ions and enhanced conductivity of protons, the composite membrane of SPEEK/MWCNTs-OH provides an effective solution to the problem of capacity fade and short lifetime.

**Figure 5:**
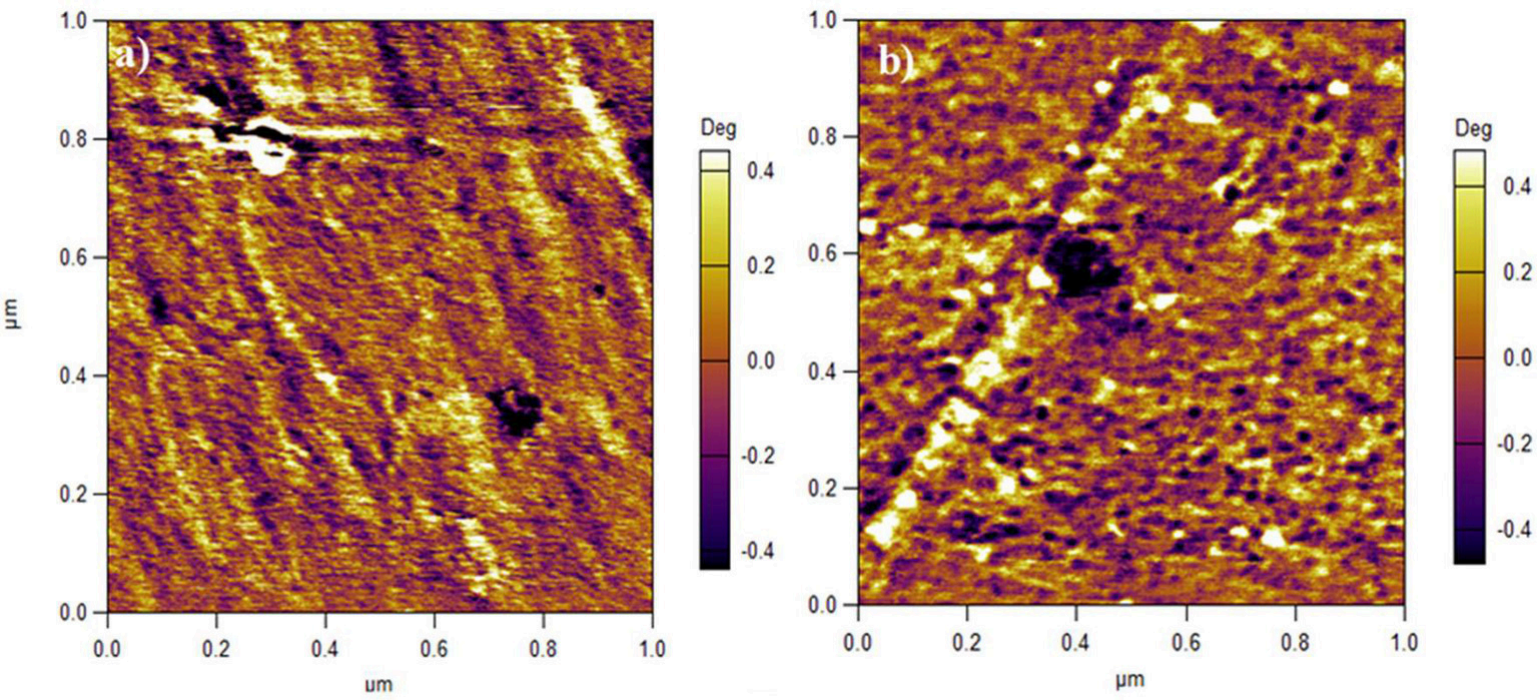
AFM images of **(a)** SPEEK membrane, **(b)** SPEEK/MWCNTs-OH composite membrane.

It is a consensus that the bright and dark domains in an AFM image represent hard hydrophobic and soft hydrophilic areas, respectively (James et al., 2000; Affoune et al., 2004). As shown in Figure 5a, the dark hydrophilic regions of SPEEK membrane form some long channels and are separated by bright hydrophobic regions. In contrast, for SPEEK/MWCNTs-OH membrane in Figure 5b, the hydrophilic regions are separated by carbon nanotubes and yield many small isolated dots. This is due to the carbon nanotubes that form C-channels, bridge S-channels, and therefore facilitate proton transport. This further echoes the mechanism described in Figure 4.

The long-term stability of a VRB system is of paramount importance in its practical application for large scale energy storage. However, it is difficult to verify the stability of membrane by cycling in a VRB cell, since the stability of carbon felt electrode under strong acid and ${\text{VO}}_{2}^{+}$ condition (e.g., the formation of V_2_O_5_ precipitates in catholyte) also leads to the VRB operation failure (Jia et al., 2010; Yuan et al., 2016). In this regard, the electrodes and electrolyte were designed to replace with refresh ones after the first charge-discharge test (i.e., the VRB cell running for 440 h). It reveals that red V_2_O_5_ precipitates formed at the edge of cathodic electrode after the test (see Figure S1 in the supporting information). Meanwhile, the XPS results show that the amount of oxygen-containing groups on carbon felt increases, such as –COOH, OH (see Figure S2 in the supporting information), resulting in an increased electrode resistance and thus a higher IR drop (see Figure S3 in the supporting information). In contrast, no increment in resistance and IR drop is observed when replacing the electrodes and electrolyte but keeping the SPEEK/MWCNTs-OH membrane (see Figure 3B). As such, the replacement was implemented in the subsequent stability test.

**Figure 6:**
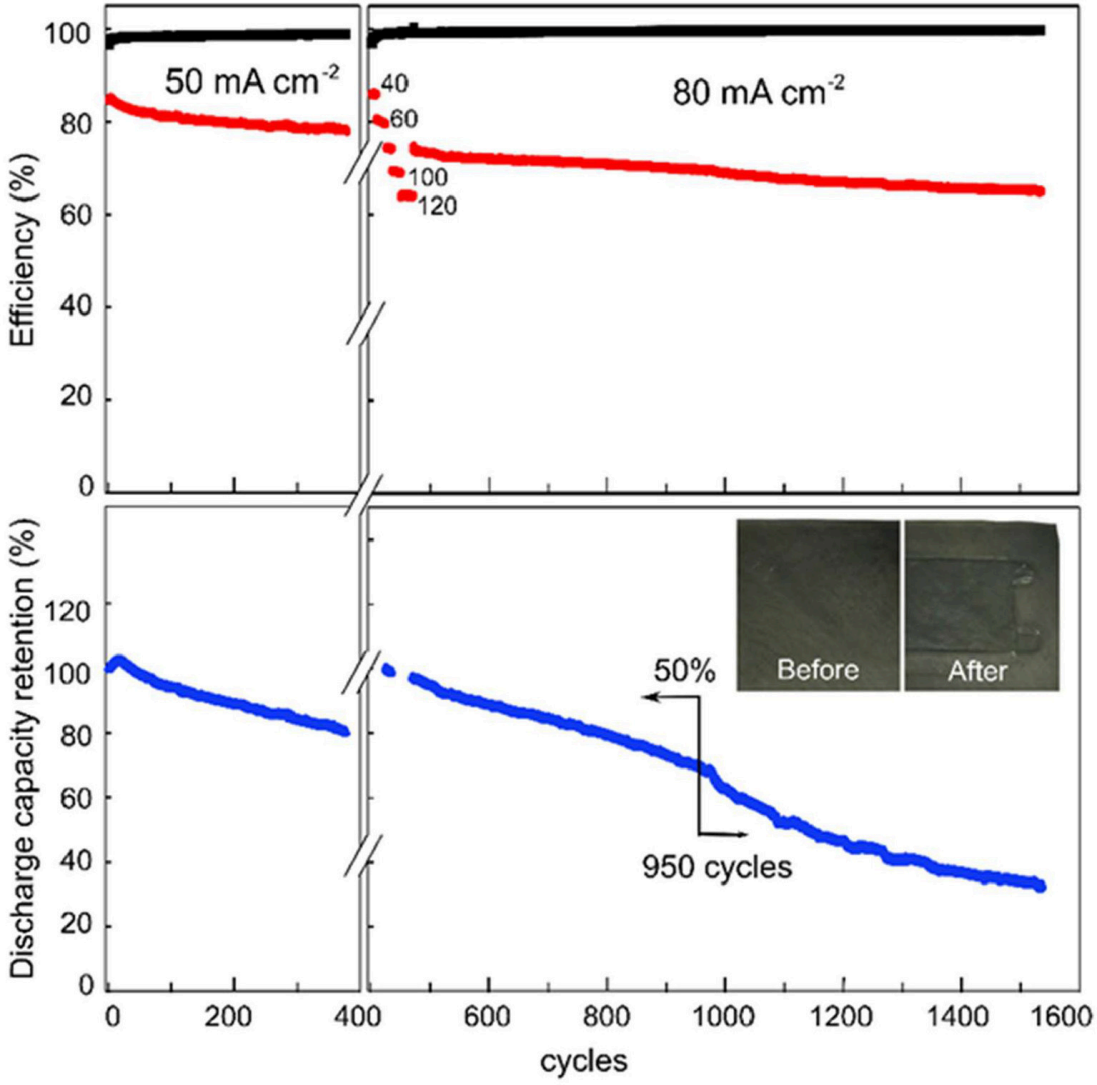
Cyclic performance of VRB with the SPEEK/MWCNTs-OH membrane: Columbic efficiency (CE, black), energy efficiency (EE, red), discharge capacity retention (DR, blue).

Figure 6 shows the cycling performance of the VRB with SPEEK/MWCNTs-OH membranes, which were initiated at a current density of 50 mA cm^−2^. After 380 cycles (i.e., 450 h), the catholyte and anolyte were refreshed while the electrodes were replaced. The cycling was continued at the following current densities sequentially: 40, 60, 80, 100, and 120 mA cm^−2^ and then back to 80 mA cm^−2^. The first cycle in the charge-discharge curves at each current density is presented in Figure 3B. Figure 6 shows that the coulombic efficiency of the cell employing the SPEEK/MWCNTs-OH membrane is over 99% during the entire cycling test (1,550 cycles, 1,030 h). The energy efficiency (EE) is about 80% at the first cycling test at 50 mA cm^−2^, while about 75% at subsequent cycling test (2nd test in VRB at 80 mA cm^−2^). The discharge retention is over 94.2% after 100 cycles and above 50% after 950 cycles. Thereafter, the retention drops substantially, because of decreased active area for chemical reactions caused by increased corrosion of carbon electrodes and deposition of ${\text{VO}}_{2}^{+}$ at the cathode. Therefore, the VRB single cell with SPEEK/MWCNTs-OH membranes exhibit an ultrahigh CE and long lifetime.

**Figure 7:**
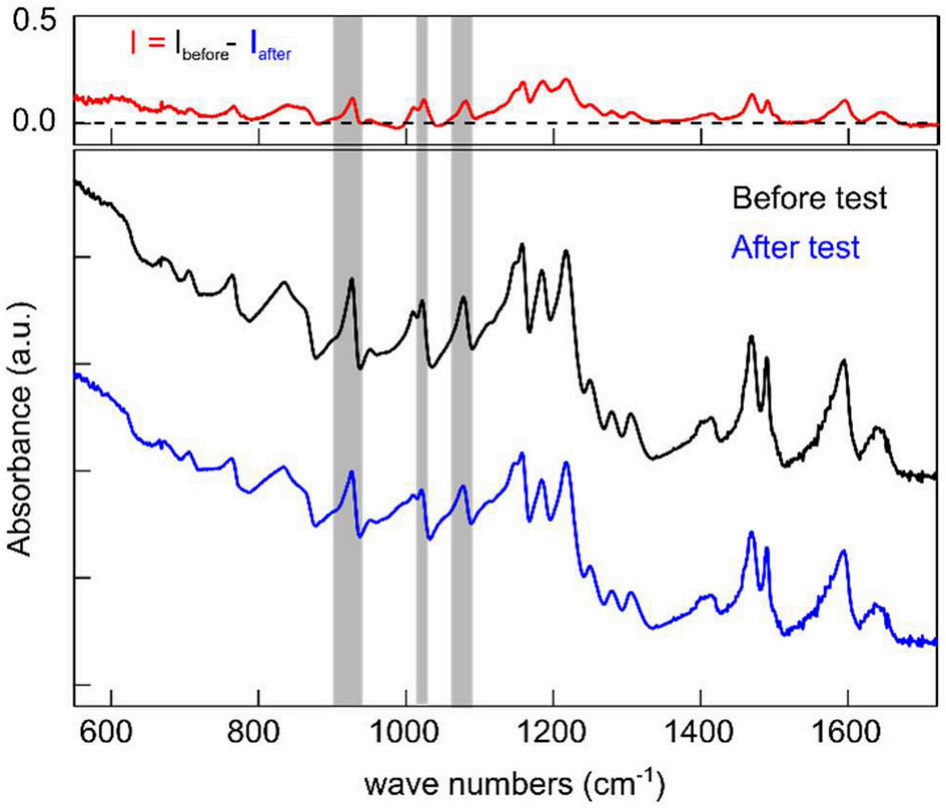
FTIR spectra of SPEEK/MWCNTs-OH membrane before (black) and after (blue) test; Top, difference spectrum I_before_-I_after_.

The FTIR spectra were measured from the SPEEK/MWCNTs-OH membrane before and after the VRB single cell test. The spectra are normalized to intensity at 877.6 cm^−1^ and therefore the difference between the normalized spectra was obtained by subtracting the spectrum of SPEEK/CNT-OH membrane after single cell test from the fresh SPEEK/CNT-OH membrane. The peaks at 926, 1,023, and 1,080 cm^−1^ are assigned to the specific S-O vibration [v(S-O)] (Swier et al., 2005), the asymmetric vibration [v_as_(SO_3_H)] and the symmetric vibration [v_s_(SO_3_H)] (Xing et al., 2004), respectively. The value of the difference spectrum I_SPEEK/MWCNTs−OH_ – I_SPEEK_ at the three peaks arising from -SO_3_H are almost zero (Figure S4), indicating almost the same amount of -SO_3_H in both membranes. This is consistent with the fact that small amount of carbon nanotubes was added to the SPEEK matrix without affecting the equivalent weight (Jalani and Datta, 2005). Peaks from -SO_3_H appear in the difference spectrum I_before_ − I_after_ of the SPEEK/MWCNTs-OH membrane indicating the degradation of the SPEEK/MWCNTs-OH membrane resulting from falling of the -SO_3_H off the surface (Figure 7). Nevertheless, the degradation is not significant as the difference between the SPEEK/MWCNTs-OH membrane before and after single cell test is within 5%. We obtained the intensity of the three peaks from -SO_3_H in both the difference spectrum I_before_ − I_after_ and the spectrum from membrane before test by integrating the corresponding regions as remarked in Figure 7 (Table S1), the ratio of the difference spectrum to the spectrum from membrane before test are ~ 5%. After a long cycling time in the single VRB cell, SPEEK/MWCNTs-OH membranes show distinctively low degradation indicating a remarkably enhanced chemical stability of the SPEEK membrane. The stability enhancement of the membrane is indeed results from the addition of carbon nanotubes.

## Conclusion

A composite membrane of SPEEK polymer blended with hydroxylated multi-walled carbon nanotubes (MWCNTs-OH) is developed toward VRB application. The membrane of SPEEK/MWCNTs-OH exhibits high mechanical strength of 61.8 MPa, suppressed permeability of vanadium ions, enhanced proton conductivity. This is attributed to the carbon nanotubes of high strength that bond strongly to the SPEEK matrix, which provides barriers for vanadium ion diffusion while additional channels for proton transport. The superior ionic selectivity, excellent proton conductivity and stability of the composite membranes ensure ultrahigh stability of over 1000 cycles in VRB cells. Such a composite membrane of SPEEK/MWCNTs-OH is of low cost, high performance and long-term stability, making it promising for the next generation large-scale VRB applications.

## Author contributions

MD design and performed the experiments. MD, XL, DY, YC, and CW prepared the samples and analyzed the data. MD, Z-SC, LS, CY, and CJ participated in interpreting and analyzing the data. All authors read and approved the final manuscript.

### Conflict of interest statement

The authors declare that the research was conducted in the absence of any commercial or financial relationships that could be construed as a potential conflict of interest.
