# Supplementary material for: SPEEK Membrane of Ultrahigh Stability Enhanced by Functionalized Carbon Nanotubes for Vanadium Redox Flow Battery
Source: Front Chem. 2018 Jul 26;6:286. doi: 10.3389/fchem.2018.00286 (PMC6094973; doi:10.3389/fchem.2018.00286)
Supplement: Supplementary file 1 [file Data_Sheet_1.docx]

**Supporting Information**

**SPEEK Membrane of Ultrahigh Stability Enhanced by Functionalized Carbon Nanotubes for Vanadium Redox Flow Battery**

Mei Ding^1^, Xiao Ling^2^, Du Yuan^3^, Yuanhang Cheng^2^, Chun Wu^1^, Zi-Sheng Chao^1^, Lidong Sun^4,5^*, Chuanwei Yan^2^, Chuankun Jia^1,2,5^*

^1^College of Materials Science and Engineering, Changsha University of Science & Technology, Changsha, 410114, China

^2^Institute of Metal Research, Chinese Academy of Sciences, Shenyang 110016, P. R. China

^3^Department of Materials Science and Engineering, Faculty of Engineering, National University of Singapore, 117576, Singapore

^4^State Key Laboratory of Mechanical Transmission, School of Materials Science and Engineering, Chongqing University, Chongqing 400044, PR China

^5^Key Laboratory of Advanced Energy Materials Chemistry (Ministry of Education), Nankai University, Tianjin 300071, China

*Corresponding author

Email: [jack2012ding@gmail.com](mailto:jack2012ding@gmail.com) (C. Jia); [lidong.sun@cqu.edu.cn](mailto:lidong.sun@cqu.edu.cn) (L. Sun)


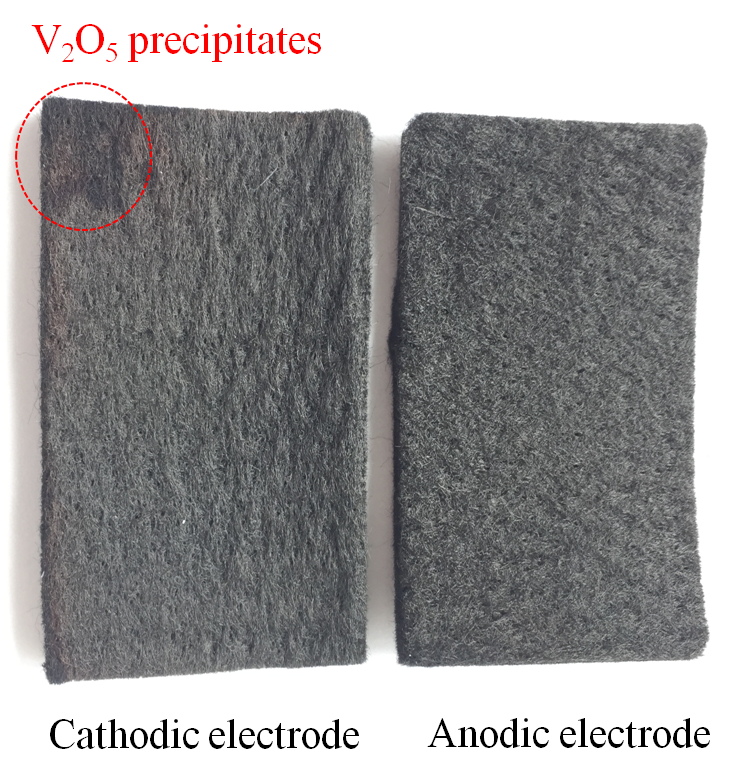


**Figure S1.** The photographs of cathodic and anodic electrodes after charging-discharging the cell for 440 h. It is visible that red V_2_O_5_ precipitates are formed at the edge of the cathodic electrode.


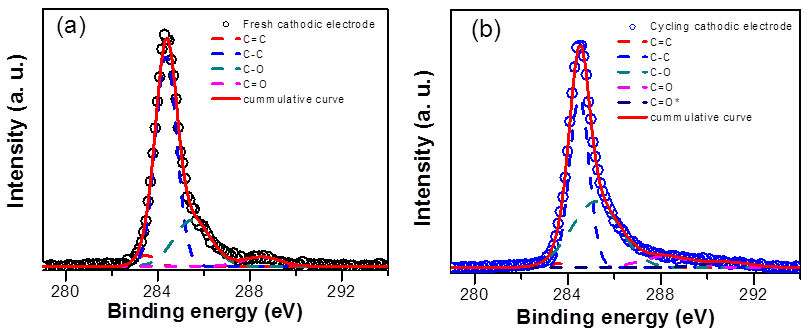


**Figure S2.** Comparison of high-resolution C1s spectra of the cathodic electrode before and after the cell cycling.


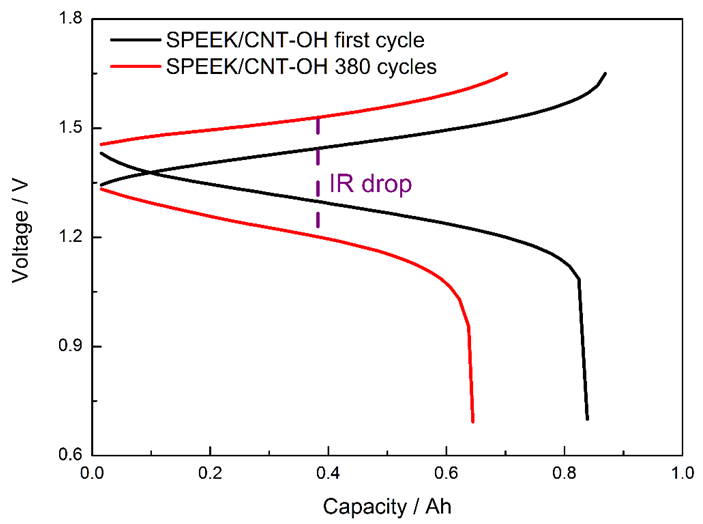


**Figure S3.** Comparison of the charge-discharge curves of VRB with SPEEK/MWCNT-OH membranes at first and 380^th^ cycle.


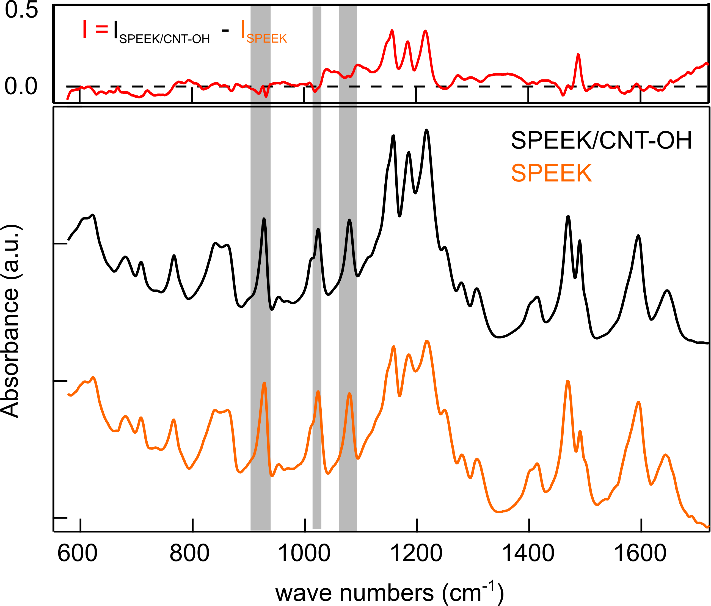


**Figure S4** FTIR spectra of SPEEN/CNT-OH membrane (black) and pure SPEEK membrane (orange). Top, difference spectrum I_SPEEK/CNT-OH_ – I_SPEEK_

**Table S1** Peak intensity of three peaks from –SO_3_H in SPEEK/CNT-OH membrane and difference spectrum I_before_-I_after_

| Peaks | Int.Dif. | Int. Bef. | Int.Dif/Int.Bef. |
| --- | --- | --- | --- |
| v(S-O) | 2.0 | 42.8 | 4.6% |
| v_as_(SO_3_H) | 1.4 | 24.4 | 5.7% |
| v_s_(SO_3_H) | 2.1 | 44.8 | 4.7% |

Int.Dif. denotes the intensity of the difference spectrum I_before_-I_after_ in Fig 7

Int.Bef. denotes the intensity of the spectrum from SPEEK/CNT-OH membrane before the single VRB cell test.
